# Global, regional and national trends in the burden of persistent pulmonary hypertension of the newborn and essentials of its management from 1993 to 2023: a scoping review

**DOI:** 10.3389/fped.2025.1502385

**Published:** 2025-06-04

**Authors:** Yan Huang, Ting Yang, Xiaoqin Liang, You Chen, Ping Zhou, Zhangbin Yu, Guichao Zhong, Lian Zhang

**Affiliations:** ^1^ Department of Neonatology, Shenzhen Baoan Women’s and Children’s Hospital, Shenzhen, Guangdong, China; ^2^ Department of Neonatology, Shenzhen People’s Hospital, (The Second Clinical Medical College, Jinan University, The First Affiliated Hospital, Southern University of Science and Technology), Shenzhen, Guangdong, China

**Keywords:** persistent pulmonary hypertension of the newborn (PPHN), neonate, epidemiology, mortality, etiologies

## Abstract

**Background:**

Persistent pulmonary hypertension of the newborn (PPHN) is a frequent neonatal emergency in the neonatal intensive care unit (*N*ICU), representing a challenging condition that has not been extensively studied. PPHNremains associated with a high mortality and morbidity.

**Objective:**

This scoping review was undertaken to provide a global overview of several key aspects: (1) the prevalence/incidence and etiologies of PPHN, (2) the mortality rate linked to PPHN during hospitalization and the primary causes of such mortality, (3) the risk factors related to PPHN, and (4) the approaches to managing PPHN. The aim of this scoping review was not to assess the methodological soundness of the identified studies, but instead to deliver a broad, comprehensive perspective on PPHN, identify gaps within the current literature, and outline potential avenues for future research. The results are anticipated to assist in developing public health strategies aimed at reducing the morbidity and mortality tied to PPHN globally.

**Methods:**

We conducted a digital search in MEDLINE and the Cochrane Library, from January 1, 1993 to December 31, 2023.We incorporated observational studies, interventional studies, and reviews that provided adequate data on the incidence/prevalence, mortality rates, predictors, etiological factors, diagnosis, and management of PPHN among the general neonatal population (age 0–28 days old). This procedure followed the guidelines set by the Preferred Reporting Items for Systematic Reviews and Meta-Analysis Extensions for Scoping Reviews (PRISMA-ScR). Additionally, we utilized the methodological framework for scoping reviews as outlined by Arksey and O'Malley, which consists of formulating the research question, conducting a search for pertinent studies, selecting the studies, organizing the data, and compiling, summarizing and reporting the findings.

**Results:**

A total of 128 research articles were collected from 27 countries categorized as either high-income or low- and middle-income countries (LMICs). The prevalence of PPHN ranges from 0.1%–8.1% in the different study populations. The highest global prevalence rates are observed in Europe and Asia, while lower prevalence rates are reported in the Americas and Africa. Neonatal infections are the leading cause of PPHN in Asia and the Americas, whereas meconium aspiration syndrome predominates in Europe. Several independent risk factors for PPHN include premature birth, male sex, ethnicity, extremes of birth weight, advanced maternal age, maternal obesity, multiple births, maternal smoking, pregestational/gestational diabetes mellitus, infectious history, caesarean delivery, antenatal drug exposure, fetal distress, APGAR score and meconium-stained amniotic fluid. The PPHN-related in-hospital mortality rate associated with PPHN ranges from 3.0%–57.9%, with the highest rates reported in Asia and the lowest in the United States of America (USA) and the United Kingdom (UK). It is advised that clinical evaluation incorporates the oxygenation index (OI) to assist in guiding medical practice.

**Conclusion:**

PPHN has a high global burden, driven by neonatal infections and meconium aspiration syndrome, particularly pronounced in LIMCs where there is a pressing need for more intensive treatments and innovative solutions, ideally supported by region-specific subsidies, to address this concerning burden.

## 1 Introduction

Persistent pulmonary hypertension of the newborn (PPHN) refers to the inability to undergo the normal transition from intrauterine circulation at birth, resulting in right-to-left shunting of deoxygenated blood both intrapulmonary and extrapulmonary at the ductus arteriosus or atrium (1, 2). It is also called continuous fetal circulation. The hallmark of PPHN is persistent and severe hypoxemia. It is primarily a state of oxygenation failure. In the most severe and untreated cases, this can lead to heart failure (3) and even death. The overall incidence of PPHN ranges from 2–4 per 1,000 live births, but the proportion of all newborns with associated with respiratory failure accompanied can be as high as 10% (4).Typically, a newborn diagnosed with PPHN is a full-term or late preterm infant who shows no significant congenital anomalies and exhibits severe respiratory failure within hours after birth, necessitating intubation and mechanical ventilation. The incidence of PPHN in this group can be as high as 5.4 per1000 live births, with mortality rates varying between 4% and 33% (5). Recent research indicates that PPHN is increasingly observed in premature infants, primarily due to the underdeveloped pulmonary vasculature. As a result, PPHN is frequently encountered as a neonatal emergency in the neonatal intensive care unit (NICU), posing significant challenges linked to high morbidity and mortality rates.

PPHN is a clinical condition marked by increased pulmonary vascular resistance following birth, caused by various factors. The primary objective of PPHN therapy is to reduce pulmonary artery pressure and improve oxygenation. Inhaled nitric oxide (iNO) is currently the sole approved pulmonary vasodilator that does not significantly decrease systemic blood pressure and specifically targets PPHN (6).Research dating back to the 1990s has shown that iNO therapy notably enhances oxygenation and lessens the necessity for extracorporeal membrane oxygenation (ECMO) in critical instances (7, 8). The standard treatments are iNO and ECMO, but they are costly and often hard to obtain in most resource-limited nations (9). Even when iNO and ECMO are available, the mortality rate remains approximately 20%. Furthermore, the overall rate of neurological dysfunction among survivors at follow-up is up to 15% (10). An observational study conducted in the USA found that 46% of surviving infants exhibited composite neurodevelopmental and audiological impairments. Among them, 13% had major neurological abnormalities, 30% had cognitive delays, and 19% had hearing loss (11). To mitigate the high mortality rate linked with PPHN, especially in settings with limited resources, it is advisable to focus on preventing PPHN through early identification of its risk factors, aiming for anticipatory, preventive, and therapeutic management of its underlying causes.

After conducting an extensive literature search, we found that there was a lack of a comprehensive summary addressing this potentially highly fatal critical neonatal condition. There are wide regional variations in PPHN mortality rates and inconsistencies in management. It is imperative that healthcare providers-including neonatologists, pediatricians, obstetricians, and fetal medicine specialists have a thorough understanding of the major causes, diagnostic approaches, and treatment options for PPHN, especially in low- and middle-income countries and regions. In this regard, large-scale data studies, such as scoping reviews, can provide robust scientific evidence that may serve as a valuable resource for shaping recommendations, guidelines, and routine clinical practice.

Consequently, we undertook this scoping review to provide a global summary of: 1. the prevalence and incidence of PPHN along with its underlying causes, 2. the mortality rate related to PPHN during hospitalization and the primary causes of death, 3. the various risk factors tied to PPHN, and 4. the treatment approaches for PPHN. The objective of this scoping review was not to assess the methodological rigor of the relevant studies identified, but to offer a comprehensive global perspective on PPHN, identify gaps in current literature, and propose avenues for future investigation. The results are anticipated to assist in the development of public health strategies aimed at reducing the morbidity and mortality linked to PPHN on a global scale.

## 2 Methods

This scoping review was carried out and presented in line with the guidelines of the Preferred Reporting Items for Systematic Reviews and Meta-Analysis Extensions specifically for Scoping Reviews (PRISMA-ScR) (12), and can be accessed as Supplementary File S1. We utilized the methodological framework for scoping reviews established by Arksey and O'Malley, which encompasses the identification of the research question, the search for pertinent studies, the selection of studies, the visualization of data, as well as the synthesis, summarization, and presentation of the findings.

### 2.1 Data source, study eligibility criteria and search strategy

A digital search was performed on MEDLINE and The Cochrane Library covering the period from January 1, 1993–December 31, 2023.We included observational studies, interventional studies and reviews that provided adequate information concerning the prevalence/incidence, mortality, predictors, etiologies, diagnosis and management of PPHN in the general neonatal population (aged 0–28 days). The search strategy used the following keywords: “neonate”, “persistent fetal circulation syndrome”, “prevalence”, “mortality”, “risk factor”, “etiologies”, “diagnosis and management”, and these were cross-referenced with the names of all countries to maximize the retrieval of relevant research articles. No restrictions related to language or geographical location were imposed on the search (Supplementary File S2). Two reviewers independently screened the titles and abstracts of the retrieved records. They subsequently examined the full texts of articles relevant to the prevalence/incidence, mortality, risk factors, etiologies, diagnosis, and management of PPHN. References from the included articles were assessed as potential sources for further studies. We excluded commentaries, expert opinions, editorial letters, case reports, and case series that involved fewer than 30 participants. To ensure more uniformity in the data concerning prevalence, etiology, and mortality of PPHN, we also discarded studies focusing on neonates selected based on the presence of PPHN-specific diseases or conditions, such as respiratory distress syndrome (RDS), transient tachypnea of the newborn (TTN), meconium aspiration syndrome (MAS), COVID-19, and neonates born to high-risk pregnancies associated with PPHN (like gestational diabetes mellitus and mothers infected with COVID-19). A comprehensive description of the search strategy can be found in Supplementary File S2.

### 2.2 Data extraction and analysis

All relevant articles were imported into EndNote 21 reference management software to eliminate duplicates. Data extraction for the study was conducted using a standardized Microsoft Excel data extraction form. Title and abstract screening and full-text review of articles were performed independently by Yan Huang and Ting Yang. Any discrepancies were resolved through discussion and consensus, with consultation from a third author (L.Z.) if necessary. Authors of individual studies may be contacted to obtain additional data or to clarify results when required. A standardized and pre-tested data extraction form was utilized by the two reviewers to independently chart the following data from each included study: bibliometric information (including the name of the first author, the country in which the study was conducted, and the year of publication), study setting, diagnostic criteria used for PPHN, sample size, proportion of males, mean gestational age (GA), GA range, prevalence or incidence, in-hospital mortality rate, risk factors, etiologies, diagnostic criteria and management of PPHN. To ensure quality assurance, all extracted and charted data were reviewed for accuracy and completeness.

### 2.3 Critical appraisal of individual sources of evidence

The evaluation of bias risk was carried out utilizing the Newcastle-Ottawa Scale (NOS) for cohort and case-control studies, the criteria established by the Agency for Healthcare Research and Quality (AHRQ) for cross-sectional studies, and modified Jadad scores specifically designed for randomized controlled trials. This evaluation was conducted by a duo of researchers, Yan Huang and Ting Yang.

## 3 Results

### 3.1 Literature search and selection of studies

**Figure 1:**
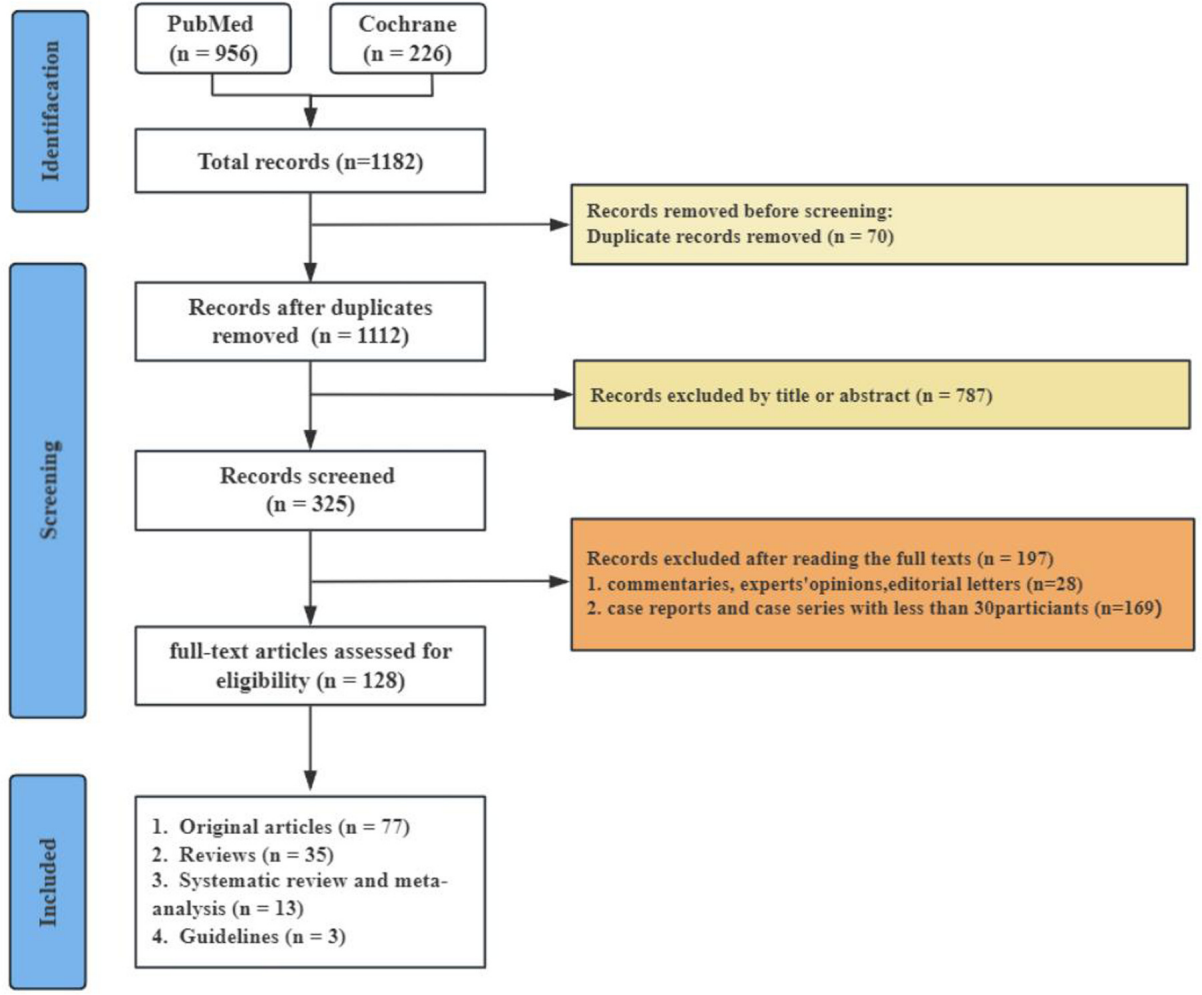
PRISMA flow diagram shows the search of the literature.

At the outset, we discovered 1,182 pertinent articles, removing 70 because of duplicates. We subsequently evaluated 1,112 articles according to established inclusion and exclusion criteria by examining the titles and abstracts, resulting in the exclusion of 787 articles. This left us with 325 articles, which were then assessed in full text. Following this full-text review, 197 articles were discarded for failing to satisfy the inclusion criteria (see Figure 1).

### 3.2 Characteristics of the selected studies

**Figure 2:**
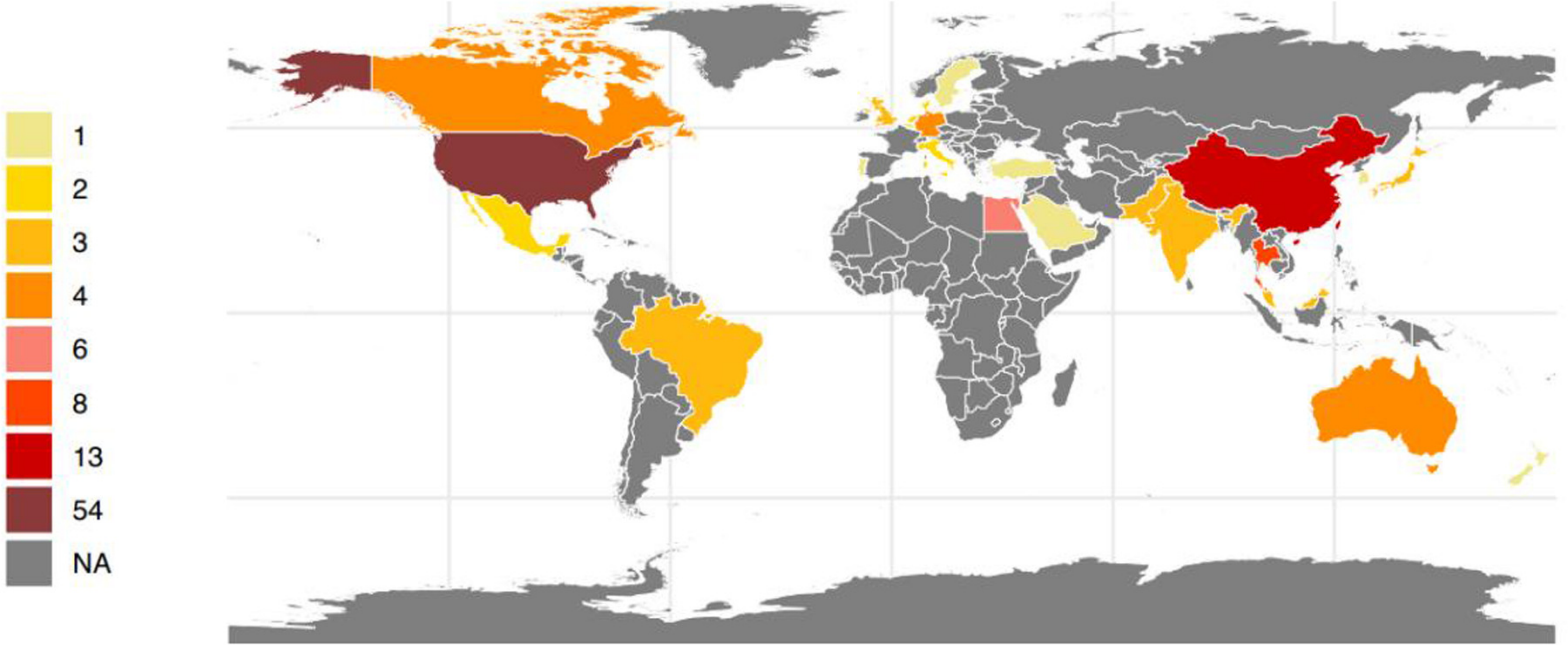
Number of research articles on persistent pulmonary hypertension of the newborn per country in the world between 1993 and 2023(created with R4.1.).

A total of 128 research articles were gathered from more than 600 centers spanning 27 different countries globally (see Figure 2). The largest proportion of these studies originated from the Americas (49.2%), with Asia following at 28.1%, Europe at 14.1%, Africa at 4.7%, and Oceania at 3.9% as detailed in Table 1. Specifically, the United States, China, and Thailand contributed 54, 13, and 8 citations, respectively (refer to Figure 2). Notably, most of the publications emerged within the previous decade, accounting for 44.5%. Additionally, there were 18 systematic reviews and 3 established guidelines (as shown in Table 1).

**Table 1:** Characteristics of the 128 papers included in the review.

Characteristic of the study	Number of papers (percentage)
Year of publication	
1993–2002	32 (25.0%)
2003–2012	39 (30.5%)
2013–2023	57 (44.5%)
Region	
Asia (9 countries)	36 (28.1%)
The Americas (4 countries)	63 (49.2%)
Europe (10 countries)	18 (14.1%)
Africa (1 countries)	6 (4.7%)
Oceania (2 countries)	5 (3.9%)
Study design	
Cross-sectional (observation)	6 (4.7%)
Case–control (observation)	24 (18.8%)
Cohort (observation)	30 (23.4%)
Clinical trial (intervention)	17 (13.3%)
Narrative or scoping review	35 (27.3%)
Systematic review and meta-analysis	13 (10.2%)
Guidelines	3 (2.3%)
Study content^a^	
Prevalence studies	44 (34.4%)
Etiological studies	34 (26.6%)
PPHN predictors or risk factors	31 (24.2%)
Diagnosis and management	64 (50.0%)

^a^ 25 papers had more than one study content.

### 3.3 Prevalence of PPHN

The prevalence of PPHN was obtained from 43 studies involving 6,128,630 neonates across 27 different countries (Table 2). In general, the worldwide prevalence of PPHN fluctuated between 0.1% and 8.1%. The prevalence varied by region as follows: Asia 0.12%–8.1% (13–28), the Americas 0.13%–5.7% (7, 11, 28–41), Europe 0.1%–8.0% (42–48), and Africa 5.0% (49, 50). More nationally representative prevalence data show that the highest prevalence of PPHN was 8.1% in Japan (19), and the lowest rate was 0.1% in a study published in Italy in 2010 (45), see Table 2.

**Table 2:** Global trends in the prevalence of PPHN**.**

First name of author, publication year	Country	Study design	Setting (number of sites if multicentre)	Sample size (*n*)	Diagnostic by Echocardiography	Diagnostic by clinical findings	Male (%)	Mean GA (weeks)	Prematurity rate (%)	CS rate (%)	Prevalence (%)	Mortality rate (%)	ROB
Rosenberg, et al. (30)	USA	Cohort	Children's Hospital	101	NR	NR	54.3	39.2 ± 1.8	NR	NR	NR	24.8	High
Wessel, et al. (31)	USA	Clinical trial	NICU	49	100	0	NR	40	NR	NR	NR	8	Low
Favilli, et al. (46)	Italy	Cross-sectional	NICU (7)	30	NR	NR	NR	NR	8.3	NR	8	22	Moderate
Davidson, et al. (7)	USA	Clinical trial	NICU (25)	155	100	0	54.8	NR	NR	61.3	NR	6.5	High
Mok, et al. (47)	UK	Case-control	NICU	32	NR	NR	NR	NR	NR	NR	NR	3.1	Moderate
Walsh-Sukys, et al. (34)	USA	Cohort	NICU (12)	202,632	72	28	58	39 ± 2	NR	51	0.19 (0.043– 0.682)	12	High
Levine, et al. (88)	USA	Cohort	Maternity	29,669	NR	NR	NR	NR	NR	17	0.13	NR	High
Su, et al. (13)	China	Case-control	NICU	40	40	60	NR	NR	NR	NR	NR	29.2	High
Lipkin, et al. (11)	USA	Clinical trial	NICU (25)	155	100	0	NR	NR	NR	NR	NR	7.1	High
Clark, et al. (35)	USA	Clinical trial	NICU	248	NR	NR	NR	NR	NR	NR	NR	10	High
Hwang, et al. (56)	South Korea	Case-control	NICU	51	NR	NR	56.9	NR	0	72.5	NR	29	Moderate
Pierce, et al. (48)	UK	Cohort	PICU	45	100	0	62.1	40	NR	44.8	NR	10.3	High
Kumar, et al. (61)	USA	Case-control	NICU	183	100	0	63.9	NR	100	NR	2	26.2	Moderate
Hernández-Díaz, et al. (36)	USA	Case-control	Epidemiology Center's Birth Defects Study	1,213	96	4	63.4	NR	16	61.3	NR	3	High
Eriksen, et al. (57)	Denmark	Cohort	NICU	130	NR	NR	NR	NR	NR	NR	NR	25	High
Peterson, et al. (37)	USA	Cohort	PICU	63	86	14	60.3	NR	NR	NR	NR	5	High
Ortiz, et al. (38)	Mexico	Cohort	NICU	38	NR	NR	61	NR	NR	NR	5.7	42.1	Moderate
Berti, et al. (49)	Italy	Cohort	NICU	31	100	0	80.6	38	NR	NR	0.1	6.5	High
Roofthooft, et al. (51)	Netherlands	Cohort	NICU	143	100	0	60.1	NR	36.4	NR	NR	31.5	High
Nakwan, et al. (14)	Thailand	Cohort	NICU (12)	41	95.1	4.9	73.2	39.4 ± 1.5	NR	NR	NR	34.1	High
Rohana, et al. (15)	Malaysia	Clinical trial	NICU	38	100	NR	NR	NR	NR	NR	NR	57.9	Low
Rocha, et al. (52)	Portugal	Cohort	NICU	6,750	NR	NR	67.9	39	20.5	65.3	1.1	32	High
Abdel Mohsen, et al. (53)	Egypt	Cohort	NICU	640	NR	NR	56.25	NR	NR	64.6	5	25	High
Razzaq, et al. (29)	Pakistan	Cross-sectional	Children's Hospital	79	100	0	72.1	NR	22.8	54.2	NR	26.6	High
Janjindamai, et al. (16)	Thailand	Cross-sectional	NICU	33	94.3	5.7	66.7	39 (30–44)	NR	69.7	NR	15.1	High
Malowitz, et al. (40)	USA	Cohort	NICU	86	NR	NR	61.6	39	NR	62.8	NR	29	High
Nakwan, et al. (17)	Thailand	Cohort	NICU	30,183	21	79	63	39.1 ± 1.6	NR	42.9	0.28	39.5	Moderate
Steurer, et al. (41)	USA	Cohort	Office of Statewide Health Planning and Development	1,781,156	17	83	58.4	38.4	15.9	57.8	0.18	7.6	High
Kamolvisit, et al. (18)	Thailand	Cohort	NICU	109	22.9	77.1	68.8	38.6	NR	45.9	NR	28.4	High
Nakanishi, et al. (19)	Japan	Cohort	NICU (202)	12,954	NR	NR	NR	NR	NR	72.5	8.1	12.5	High
Maneenil, et al. (20)	Thailand	Cross-sectional	NICU	40	97.5	2.5	67.5	38	NR	NR	NR	12.5	Moderate
Steurer, et al. (42)	USA	Case-control	Office of Statewide Health Planning and Development	3,974,536	NR	NR	NR	NR	NR	NR	0.2	7.3	High
Liu, et al. (21)	China	Cohort	NICU	115	NR	NR	55.7	34.9	52.2	66.1	NR	12.2	High
Berger-Caron, et al. (43)	Canada	Cohort	NICU/PICU	43	NR	NR	58	36 ± 6	NR	NR	NR	53.5	High
Nakwan, et al. (22)	Thailand/India/Japan/Kuwait/Pakistan/Singapore	Cross-sectional	NICU (7)	369	84.8	15.2	61.2	36.1 ± 4.1	<34w (16.3%)	NR	0.12–0.46	20.1 (11.8–36.4)	High
Arshad, et al. (23)	Pakistan.	Cohort	Pediatric Department	122	88.5	11.5	66.3	NR	36	57.4	NR	21.3	Moderate
Mat Bah, et al. (24)	Malaysia	Cross-sectional	NICU (22)	82,915	100	0	60	38.8 ± 1.7	19.5	57.4	0.207	16.4	Moderate
Aleem, et al. (44)	USA	Cohort study	NICU (164)	2,743	NR	NR	58.7	38	20.1	NR	NR	6.7	High
Lin, et al. (25)	China	Case-control	NICU	203	NR	NR	49.8	NR	43.8	64.5	NR	33	High
Jastania, et al. (26)	SAU	Cohort	NICU	56	100	0	67.9	NR	46.4	NR	NR	46.4	Moderate
Qian, et al. (27)	China	Cohort	NICU	105	100	0	66.7	38.4 ± 1.9	NR	NR	NR	24.8	High
Kamran, et al. (28)	Pakistan	Cross-sectional	Pediatric Cardiology Department	82	100	0	52.4	38.28 ± 1.13	NR	36.6	NR	7.3	Moderate
Dyess, et al. (45)	USA	Cohort	NICU (28)	224	89	11	58	27	100	77	NR	37	High

NR, not reported; GA, gestational age; CS, cesarean section; ROB, risk of bias.

### 3.4 Etiologies of PPHN

In our analysis of the causes of PPHN within the subgroup, we assessed information from 34 studies including 4,170,496 neonates from 17 countries (Table 3). Globally, the leading etiologies of PPHN were neonatal infection (*n* = 3,709), MAS (*n* = 2,918), idiopathic pulmonary hypertension (*n* = 1,909) and RDS (*n* = 1,205) (Table 3). The leading etiologies of PPHN varied regionally. Neonatal infections were the leading etiology of PPHN in Asia, the Americas and Africa,whereas MAS was the leading etiology in Europe, as indicated at the conclusion of Table 3.

**Table 3:** Mortality related to PPHN per its etiologies at national and regional levels.

First name of author, publication year	Country	Sample size (*n*)	PPHN cases (*n*)	Number of cases of the different etiologies of PPHN (*n*)							ROB
				RDS/HMD	MAS	Infection	Perinatal asphyxia	CDH/lung hypoplasia	IPH	Others	
Rosenberg, et al. (30)	USA	101	101	9	23	0	0	21	0	12	High
Wessel, et al. (31)	USA	49	49	1	22	12	0	0	11	2	Low
Kinsella, et al. (72)	USA	205	205	70	58	23	0	34	0	20	Low
Davidson, et al. (7)	USA	155	155	17	86	43	0	0	29	27	High
Mok, et al. (47)	UK	32	32	0	17	2	3	0	10	0	Moderate
Cornfield, et al. (33)	USA	38	38	8	8	10	0	9	3	0	High
Walsh-Sukys, et al. (34)	USA	71,558	385	50	159	54	0	54	66	2	High
Ellington, et al. (10)	USA	83	83	0	41	0	0	0	0	0	Low
Torielli, et al. (73)	USA	78	78	10	25	21	0	12	7	3	High
Clark, et al. (35)	USA	248	248	17	74	0	0	20	44	0	High
Pierce, et al. (48)	UK	45	29	2	13	5	1	1	2	5	High
Hwang, et al. (56)	South Korea	51	51	11	17	3	0	10	10	0	Moderate
Hernández-Díaz, et al. (36)	USA	1,213	377	NR	179	238	0	0	0	0	High
Eriksen, et al. (57)	Denmark	130	85	0	0	1	0	5	20	59	High
Berti, et al. (49)	Italy	31	31	3	7	3	5	0	4	4	High
Uslu, et al. (50)	Turkey	65	65	14	30	7	7	0	5	2	Low
Nakwan, et al. (14)	Thailand	41	41	0	21	3	0	3	4	1	High
Rocha, et al. (52)	Portugal	6,750	78	3	10	19	4	19	0	21	High
Byers, et al. (39)	USA	88	88	0	21	19	16	0	0	31	High
Abdel Mohsen, et al. (53)	Egypt	640	32	6	16	24	14	4	0	13	High
Razzaq, et al. (29)	Pakistan	79	79	11	28	23	0	0	0	0	High
Yao, et al. (55)	China	106	66	22	11	13	14	0	0	0	High
Janjindamai, et al. (16)	Thailand	33	33	0	18	14	0	0	1	0	High
Nakwan, et al. (17)	Thailand	30,183	119	1	65	41	0	3	1	0	Moderate
Kamolvisit, et al. (18)	Thailand	109	109	5	67	23	0	9	3	0	High
Maneenil, et al. (20)	Thailand	40	40	0	14	15	0	0	3	8	Moderate
Steurer, et al. (42)	USA	3,974,536	7,847	660	1,628	2,598	0	470	1,592	899	High
Liu, et al. (21)	China	115	115	53	10	44	0	0	0	0	High
Berger-Caron, et al. (43)	Canada	43	43	0	5	0	0	6	11	21	High
Nakwan, et al. (22)	Thailand/India/Japan/Kuwait/Pakistan/Singapore	369	369	79	89	96	13	24	46	35	High
Arshad, et al. (23)	Pakistan.	122	122	23	52	42	48	6	18	0	Moderate
Mat Bah, et al. (24)	Malaysia	82,915	195	14	64	50	19	20	15	13	High
Lin, et al. (25)	China	203	203	108	23	250	78	8	0	96	High
Wei, et al. (85)	China	42	42	8	17	13	0	0	4	0	High
Total (*n*)		41,70,496	11,633	**1,205**	**2,918**	**3,709**	222	738	**1,909**	1,274	
**Regions**	**Number of studies**	**Sample of the studies (n)**	**Total PPHN cases (n)**	**RDS/HMD**	**MAS**	**Infection**	**Perinatal asphyxia**	**CDH/lung hypoplasia**	**IPH**	**Others**	
Americas	13	40,48,395	9,697	842	2,329	3,018	16	626	**1,763**	1,017	
Asia	14	114,408	1,584	**335**	**496**	**630**	172	83	105	153	
Europe	6	7,053	320	22	77	37	20	25	41	91	
Africa	1	640	32	6	16	24	14	4	0	13	

Others (*n*): anomalies of the respiratory system = 508, CHD = 82, pneumothorax = 58, TTN = 43, pulmonary hemorrhage = 13, small for gestational age = 6, severe hypotension with shock = 1, hydrops fetalis = 12, Related to trisomy 21 = 157, Vena galena aneurism = 1, Lung disease = 45, Hypoglycemia = 8, adenomatoid cystic pulmonary malformation = 2, placental disruption = 2, Polycythemia = 35, Intrathoracic neuroblastoma = 1, Down syndrome = 4, Atelectasis = 9, Potter syndrome = 1, Idiopathic pulmonary arteriolar calcification = 1, Arterial pulmonary thrombosis = 1, Arterial pulmonary thrombosis = 2, Large for Gestational Age = 31, Renal agenesis = 2, Cystic kidney = 6.

Bolded values indicate the highest counts in their respective categories.

RDS, respiratory distress syndrome; HMD, hyaline membrane disease; MAS, meconium aspiration syndrome; CDH, congenital diaphragmatic hernia; IPH, idiopathic pulmonary hypertension; TTN, transient tachypnoea of the newborn; ROB, risk of bias.

### 3.5 PPHN-related death predictors, mortality rate and etiologies of death

Predictors associated with mortality in PPHN included a minimum PaO2/FIO2 ratio of less than 0.3 (OR = 8.26, 95% CI 1.78–38.41), a urine output of below 1.0 ml/kg/h within the initial 12 h (OR = 7.30, 95% CI 1.16–45.95), and a lowest mean blood pressure under 30 mm Hg (OR = 5.58, 95% CI 1.15–26.99). Infants presenting with SNAP-II score of 43 or higher faced the highest rist of mortality, reflected by an odds ratio (OR) of 10.00 (95% CI 1.03–97.50). Each one-point increase in the SNAP score was linked to a 1.04 increase in the odds of mortality (95% CI 1.01–1.07, *P* < 0.01) (14). The major risk factors for PPHN across various age groups encompassed male sex, cesarean deliveries, MAS, and RDS. Notably, RDS (RR = 5), birth asphyxia (RR = 2.5), and male gender (RR = 2) correlated with a heightened mortality risk in preterm infants in comparison to their term and post-term counterparts (51). A multicenter study in Malaysia identified that lower APGAR scores at five minutes and impaired cardiac function were linked to poorer outcomes. Key independent mortality risk factors comprised reverse flow detected in the descending aorta (OR = 15.91, 95% CI 5.64–44.92), APGAR scores of five or less at 5 min (OR = 6.72, 95% CI 2.04–22.15), and idiopathic pulmonary hypertension (OR = 6.46, 95% CI 1.52–27.43) (24). On a different note, pneumothorax (adjusted HR = 2.07, 95% CI 1.09–3.93, *p* = 0.03) and acute kidney injury (AKI) (adjusted HR = 2.99, 95% CI 1.59–5.61, *p* < 0.01) were significantly related to a higher likelihood of death. Conversely, conditions such as ventilator-associated pneumonia (VAP) (adjusted HR = 0.32, 95% CI 0.15–0.67) and the administration of total parenteral nutrition (TPN) (adjusted HR = 0.22, 95% CI 0.10–0.50) were linked to reduced mortality rates (17). Variations in plasma concentrations of atrial natriuretic peptide (ANP), endothelin-1 (ET-1) and von Willebrand factor (vWF) could indicate pulmonary artery systolic pressure (PASP) in newborns suffering from PPHN throughout their treatment. Ongoing assessment of these biomarkers may facilitate an evaluation of PPHN severity and direct appropriate treatment approaches (52).

Worldwide, the in-hospital mortality rates linked to PPHN fluctuated between 3.0 and 57.9%,showing regional variations as follows: Asia 7.3–57.9% (13–24, 28), the Americas 3.0–53.5% (7, 11, 28–40), Europe 3.1%–32% (42–48, 53) and Africa 25% (49). At the national level,this mortality rate was highest in Malaysia 57.9% (15) and Canada 53.5% (54) and lowest in the USA 3% (36)and the UK 3.1% (43), see Table 2. A California report focused on late preterm and term infants indicated that the one-year mortality associated with PPHN was significantly influenced by the underlying causes, with the highest rates seen in infants with other congenital respiratory anomalies (32%), followed by congenital diaphragmatic hernia (CDH) at 25.0%, respiratory distress syndrome (RDS) at 6.9%, and other causes at 8% (40).

Additionally, in a separate analysis of PPHN-related mortality, we evaluated data from 20 studies encompassing 5,942, 464 neonates across 13 countries (Table 4). Globally, the predominant causes of PPHN-related fatalities included neonatal infections (*n* = 334), CDH/lung hypoplasia (*n* = 269), MAS (*n* = 162) and RDS (*n* = 118). These trends showed minimal regional variation, with CDH/lung hypoplasia being the primary cause of PPHN-related in-hospital mortality in the Americas. In Asia, neonatal infections led as the primary cause of death. Conversely, fewer PPHN-related deaths were studied in Europe and Africa (Table 4).

**Table 4:** Mortality related to PPHN per its etiologies at national and regional levels.

First name of author, publication year	Country	Sample size (*n*)	PPHN cases (*n*)	PPHN non-survivors(*n*)	Number of non-survivors per etiologies of PPHN (*n*)												ROB
					Alveolar capillary dysplasia	Hemo-rrhage	Pneumo-thorax	RDS/HMD	MAS	Infection	Perinatal asphyxia	CDH/pulmonary hypoplasia	IPH	CHD	TTN	Others	
Wessel, et al. (31)	USA	49	49	4	3	1	0	0	0	0	0	0	0	0	0	0	High
Davidson, et al. (7)	USA	155	155	10	1	0	5	0	0	3	1	0	0	0	0	0	High
Mok, et al. (47)	UK	32	32	1	0	0	0	0	1	0	0	0	0	0	0	0	Moderate
Walsh-Sukys, et al. (34)	USA	71,558	385	46	0	0	0	5	10	5	0	21	0	0	0	5	High
Clark, et al. (35)	USA	248	248	24	0	7	0	0	0	0	0	12	0	0	0	5	High
Pierce, et al. (48)	UK	45	29	3	0	0	0	0	0	0	0	3	0	0	0	0	High
Berti, et al. (49)	Italy	31	31	2	0	1	0	0	0	1	0	0	0	0	0	0	High
Nakwan, et al. (14)	Thailand	41	41	14	0	0	0	0	6	2	0	3	1	0	2	0	High
Abdel Mohsen, et al. (53)	Egypt	640	32	8	0	0	1	0	0	3	4	0	0	0	0	0	High
Razzaq, et al. (29)	Pakistan	79	79	21	0	0	0	7	10	10	0	0	0	0	0	0	High
Janjindamai, et al. (16)	Thailand	33	33	5	0	0	3	0	0		0	0	0	0	0	2	High
Nakwan, et al. (17)	Thailand	30,183	119	47	0	0	0	0	23	18	0	2	1	0	3	0	Moderate
Steurer, et al. (41)	USA	17,81,156	3,277	248	0	0	0	17	10	15	0	62	7	0	0	99	High
Kamolvisit, et al. (18)	Thailand	109	109	31	0	0	0	2	16	7	0	5	1	0	2	0	High
Maneenil, et al. (20)	Thailand	40	40	5	0	0	0	0	0	0	0	0	2	0	0	3	Moderate
Steurer, et al. (42)	USA	39,74,536	7,847	571	0	0	0	30	59	149	0	130	52	0	0	151	High
Nakwan, et al. (22)	Thailand/India/Japan/Kuwait/Pakistan/Singapore	369	369	76	0	0	0	15	15	23	0	16	1	0	1	5	High
Mat Bah, et al. (24)	Malaysia	82,915	195	32	0	0	0	2	5	3	3	11	5	0	0	2	Moderate
Lin, et al. (25)	China	203	203	67	0	31	9	40	7	95	33	4	0	15	0	0	High
Wei, et al. (85)	China	42	42	1	0	0	0	0	0	0	0	0	0	0	0	1	High
Total (n)		59,42,464	13,315	1,216	4	40	18	118	162	334	41	269	70	15	8	273	
**Regions**	**Number of studies**	**Sample size (*n*)**	**PPHN cases (*n*)**	**PPHN non-survivors (*n*)**	**Number of non-survivors per etiologies of PPHN (*n*)**												
					**Alveolar capillary dysplasia**	**Hemo-rrhage**	**Pneumo-thorax**	**RDS/HMD**	**MAS**	**Infection**	**Perinatal asphyxia**	**CDH/pulmonary hypoplasia**	**IPH**	**CHD**	**TTN**	**Others**	
**Americas**	6	58,27,702	11,961	903	4	8	5	52	79	172	1	225	59	0	0	260	
**Asia**	10	1,14,014	1,230	299	0	31	12	66	82	158	36	41	11	15	8	13	
**Europe**	3	108	92	6	0	1	0	0	1	1	0	3	0	0	0	0	
**Africa**	1	640	32	8	0	0	1	0	0	3	4	0	0	0	0	0	

Others (*n*): Pulmonary anomaly = 191, Related to trisomy 21 = 2, Others = 39 (birth asphyxia, cystic kidney disease, hydrops fetalis, interstitial emphysema, leukemia, polycythemia, renal agenesis and dysgenesis, trisomy).

RDS, respiratory distress syndrome; HMD, hyaline membrane disease; MAS, meconium aspiration syndrome; CDH, congenital diaphragmatic hernia; IPH, idiopathic pulmonary hypertension; TTN, transient tachypnoea of the newborn; ROB, risk of bias.

### 3.6 Risk factors associated with PPHN

#### 3.6.1 Fetal factor

Gestational age (GA) Numerous studies have consistently indicated that among the various causes of PPHN, premature newborns experience significantly higher rates than those born at term (35, 40, 55, 56). A retrospective, multicenter cohort study from Japan revealed that the prevalence of PPHN was 8.1% (95% CI 7.7%–8.6%), showing an upward trend each year, with an increased prevalence correlating with decreasing GA: for infants born at 22 weeks, the rate was 18.5% (ranging from 15.2%–22.4%), as opposed to 4.4% (ranging from 3.8%–5.2%) for those delivered at 27 weeks (19). A meta-analysis found that a gestational age of less than 37 weeks (OR = 4.34, CI 1.64–11.5) was one of the three key risk factors (57). Likewise, a prolonged gestational age (≥ 42 weeks) has become increasingly acknowledged as a significant risk element for PPHN (35, 49, 57).

##### 3.6.1.1 Birth weight

In comparison to infants within the 10th and 90th percentiles of birth weight related to gestational age, the risk was heightened for those exceeding the 90th percentile. Both large and small for gestational age classifications were independently linked to PPHN (35, 40, 49). Low birth weight was significantly associated with death in preterm neonates with PH (58).

##### 3.6.1.2 Gender

Gender The male sex has frequently been identified as an independent risk factor for PPHN (35, 40, 51). Additionally, male infants have shown a significant correlation with increased mortality in preterm neonates with PH (58). This was confirmed in the meta-analysis (OR = 1.84, 95% CI 1.28–2.63) (57).

##### 3.6.1.3 Amniotic fluid (meconium stained amniotic fluid/MAS/oligohydramnios)

Meconium aspiration syndrome (MAS) has been reported as the predominant risk factor across multiple studies (22, 49, 51), a conclusion further supported by a corresponding meta-analys (57). Oligohydramnios has also been linked to the onset of PPHN in preterm infants (58).

##### 3.6.1.4 APGAR scores/birth asphyxia

APGAR scores of 5 or lower at 5 min have been recognized as a standalone risk factor for mortality in PPHN, showing an adjusted odds ratio of 6.7 (95% CI 2.04–22.15) (24). In a similar vein, additional research has uncovered a link between low Apgar scores and a heightened risk of mortality (49, 51, 55, 58). Moreover, there is an increased mortality risk associated with birth asphyxia in preterm infants when compared to both term and postterm infants (51). A meta-analysis of 22 primary studies (*n* = 7,937 case records and 2,613,072 control cases), published by October 29, 2021, revealed a combined odds ratio of 3.9 (95% CI 2.87–5.31) relating perinatal asphyxia to the risk of PPHN (57).

#### 3.6.2 Maternal risk factor

##### 3.6.2.1 Maternal age

Advanced age in mothers has consistently been identified as a contributing factor to mortality related to PPHN (40, 57).

##### 3.6.2.2 Maternal obesity

Numerous studies have indicated that obesity in mothers poses a risk factor for PPHN (35, 40, 55).

##### 3.6.2.3 Maternal smoking

Research has revealed a notable risk of PPHN associated with maternal smoking both before and during pregnancy, with an odds ratio of 4.85 (95% CI 1.98–11.9) (57, 59). However, the study by Araujo did not report any interaction between smoking and PPHN (55).

##### 3.6.2.4 Ethnicity

Research by Hernández-Díaz illustrated that being of black or Asian descent in mothers was linked to a heightened risk for PPHN (35). In contrast, Hispanic ethnicity was protective against PPHN (40).

#### 3.6.3 Obstetrical risk factors

##### 3.6.3.1 Gestational diabetes mellitus

Several studies without controversy have demonstrated that maternal diabetes was more common in all etiologies of PPHN (35, 40, 55). Newborns born to mothers with chronic diabetes mellitus, gestational diabetes mellitus (GDM) and pregestational diabetes mellitus (PGDM) face an elevated risk of PPHN (OR = 3.61, 95% CI 2.02–6.45) (57).

##### 3.6.3.2 Chorioamnionitis

In a Japanese cohort study, clinical chorioamnionitis and premature rupture of membranes were correlated with PPHN (19).

##### 3.6.3.3 Multiple gestation

A study involving late preterm and term infants in California found that multiple births offered a protective effect against PPHN (40).

##### 3.6.3.4 Mode of delivery

Numerous studies have emphasized that the likelihood of PPHN post-cesarean section(CS) is significantly higher compared to that following vaginal delivery (35, 51, 60). In a retrospective cohort study, the raw relative risk (RR) of PPHN in elective cesareans performed before labor, vs. intended vaginal deliveries, was 2.0 (95% CI 1.3–3.1). In comparing elective cesareans to spontaneous labor resulting in vaginal deliveries, the RR of PPHN was observed to be 3.4 (95% CI 2.1–5.5). When gestational age at birth (less than vs. equal to or more than 37 weeks) was taken into account, the adjusted RRs for these delivery groups were 2.2 (95% CI 1.4–3.4) and 3.7 (95% CI 2.3–6.1,respectively. The rate of PPHN in the elective cesarean cohort was recorded as 6.9 per 1,000 deliveries. To prevent a single occurrence of PPHN in this population, it would be necessary to avoid 387 cesarean sections (number needed to harm, 95% CI 206.8–3,003.1) (61).

##### 3.6.3.5 Antenatal drugs

As early as 1996, the intake of nonsteroidal anti-inflammatory drugs and aspirin by pregnant women, along with the reasons for their consumption, appeared to be linked to a heightened risk of PPHN (62). In 2006, advisories from Health Canada and the US Food and Drug Administration cautioned healthcare providers regarding a potential connection between the maternal use of selective serotonin reuptake inhibitors (SSRIs) during pregnancy and the incidence of PPHN. A large cohort study, involving roughly 30,000 women who took SSRIs during pregnancy in five Nordic nations, was carried out to assess whether SSRIs used by mothers elevate the risk of PPHN and to explore if this risk varies among different SSRIs. The findings suggested that while the overall risk of PPHN is comparatively low, the likelihood increases more than twofold when SSRIs are used in the later stages of pregnancy. This heightened risk appears to be a class effect, with the increased likelihood of PPHN being of similar magnitude across various SSRIs, including sertraline, citalopram, paroxetine, and fluoxetine (63). Nonetheless, high-quality evidence from a meta-analysis encompassing seven studies released before January 2014 indicated that the absolute risk difference for developing PPHN after being exposed to SSRIs in late pregnancy ranged from 2.9–3.5 per 1,000 infants. Consequently, it is estimated that 286–351 women would need to be treated with an SSRI during the late stages of pregnancy to yield an average of one additional PPHN case. Clinically, the absolute risk of PPHN remained low, and the increase in risk seems more modest than earlier research suggested (64). This finding was further validated by a meta-analysis of 11 studies (*n* = 156,978 women) published by January 2019, which revealed that the use of SSRIs or serotonin-norepinephrine reuptake inhibitors during pregnancy was associated with a greater risk of PPHN (OR = 1.82, 95% CI 1.31–2.54, *I*^2^ = 72%). Based on the results, sertraline appears to possess the lowest potential risk for PPHN when compared to other SSRIs, indicating it could offer the most favorable safety profile for use during pregnancy in this context. Additional research is required to validate these results (65).

### 3.7 Essentials of the diagnosis and management of PPHN

#### 3.7.1 Diagnosis of PPHN

##### 3.7.1.1 History

A detailed history guides in identifying the aforementioned maternal, fetal and/or obstetrical risk factors associated with the common etiologies of PPHN.

##### 3.7.1.2 Clinical diagnosis

In the majority of included studies, the diagnosis of PPHN was primarily based on clinical or echocardiographic evidence of pulmonary hypertension (PH). The most frequent PPHN definition cited by 9 authors (14, 17, 18, 20, 22, 27, 33, 38, 66) was the presence of at least two of the following conditions: (1) documented pulmonary hypertension, as defined by echocardiographic evidence of elevated pulmonary pressure (right to left or bidirectional shunt), (2) a pre-to-post ductal partial pressure of oxygen gradient of 10–20 mmHg (1 mmHg = 0.133 kPa), and (3) a pulse oximetry oxygen saturation (SpO2) gradient ≥5%.

#### 3.7.2 Assessment of the severity of PPHN

Various clinical scores have been suggested to classify the severity of PPHN into three categories: mild, moderate and severe. One score is determined based on pulmonary artery pressure values obtained through echocardiography, while another score is derived from the OI value, which is calculated using the formula: OI value = FiO2  ×  Mean airway pressure cmH2O/Arterial blood PO2 mmHg. Two authors categorized pulmonary hypertension as mild if the estimated pulmonary artery systolic pressure (PASP) was under 40 mmHg, moderate if ranged from 40–60 mmHg, and severe if it exceeded 60 mmHg (48, 50). In contrast, another author classified pulmonary hypertension as mild for PSAP values below 50 mmHg, moderate for values between 51 and 70 mmHg, and severe for values above 70 mmHg (23). An OI value of less than 15 was deemed mild PPHN, while values from 15–25 were classified as moderate PPHN, values between 25 and 40 as severe, and any value over 40 as very severe PPHN (47, 58).

#### 3.7.3 Management of PPHN

The principles of treatment for confirmed PPHN include: 1. Ensuring adequate lung recruitment and alveolar ventilation with gentle ventilation, 2. Supporting of cardiovascular function, 3. Correcting severe acidosis while maintaining optimal blood pH and avoiding over-alkalinization, 4. Application of pulmonary vasodilators, and 5. Application of ECMO. This study summarizes the main treatments for PPHN in different studies and regions, see Table 5.

**Table 5:** Essentials management of PPHN.

First name of author, publication year	Country	Sample size (*n*)	PPHN cases (*n*)	HFV (%)	Surfac-tant (%)	Vasodilator (%)						ECMO (%)	Inotropes (%)	Tolazoline (%)	Sedation (%)	Paraly-sis (%)	Hydrocor-tisone (%)	DMV (days)	DSO (days)	ROB
						iNO (%)	Sildenafil (%)	Bosen-tan (%)	Milrino-ne (%)	Prosta-glandin (%)	MgSO_4_ (%)									
Rosenberg, et al. (30)	USA	101	101	NR	NR	86.1	NR	NR	NR	NR	NR	16.8	NR	NR	NR	NR	NR	NR	NR	High
Wessel, et al. (31)	USA	49	49	NR	NR	53.1	NR	NR	NR	NR	NR	33	NR	NR	NR	NR	NR	9	13	Low
Wood, et al. (32)	USA	29	29	96.6	79.3	100	NR	NR	NR	NR	NR	44.8	93.1	NR	NR	NR	NR	NR	NR	Low
Mok, et al. (47)	UK	32	32	25	NR	87.5	NR	NR	NR	NR	NR	NR	NR	NR	NR	NR	NR	NR	NR	Moderate
Walsh-Sukys, et al. (34)	USA	71,558	385	39 (0%–76.3%)	36 (12%–71%)	8	39 (13%– 81%)	NR	NR	NR	NR	34 (0%– 85%)	84 (46%–100%)	53	94 (77%–100%)	73 (33%–98%)	NR	NR	NR	High
Ellington, et al. (10)	USA	49	49	47	NR	51.8	NR	NR	NR	NR	NR	31.3	NR	NR	NR	NR	NR	NR	NR	Low
Clark, et al. (35)	USA	248	248	NR	NR	50.8	NR	NR	NR	NR	NR	39.5	NR	NR	NR	NR	NR	NR	NR	High
Pierce, et al. (48)	UK	29	29	NR	62.1	89.7	NR	NR	NR	NR	NR	NR	NR	NR	NR	NR	NR	3	NR	High
Hwang, et al. (56)	South Korea	51	51	76.5	35.3	100	NR	NR	NR	NR	NR	NR	90.2	NR	NR	92.2	NR	NR	NR	Moderate
Hernández-Díaz, et al. (36)	USA	1,213	377	70	NR	67.1	NR	NR	NR	NR	NR	NR	NR	NR	NR	NR	NR	NR	NR	High
Peterson, et al. (37)	USA	63	63	52	NR	67	NR	NR	NR	NR	NR	14	70	NR	NR	NR	NR	NR	NR	High
Ortiz, et al. (38)	Mexico	217	38	NR	NR	NR	NR	NR	NR	NR	NR	NR	55.3	NR	63.2	52.6	NR	NR	NR	Moderate
Roofthooft, et al. (51)	Netherlands	143	143	NR	NR	67.4	NR	NR	NR	NR	NR	NR	NR	NR	NR	NR	NR	NR	NR	High
Nakwan, et al. (14)	Thailand	41	41	66.7	NR	0	NR	14.8	NR	55.6	NR	NR	NR	NR	NR	NR	NR	NR	NR	High
Rocha, et al. (52)	Portugal	6,750	78	NR	30.7	24.3	15.3	NR	NR	NR	NR	1.2	73	NR	91	NR	NR	7 (1–114)	NR	High
Abdel Mohsen, et al. (53)	Egypt	640	32	NR	NR	NR	50	NR	NR	NR	31.25	NR	NR	NR	NR	37.5	NR	NR	NR	High
Nakwan, et al. (17)	Thailand	30,183	119	80.7	NR	31.1	69.7	NR	NR	80.7	NR	NR	100	NR	100	NR	NR	NR	NR	Moderate
Kamolvisit, et al. (18)	Thailand	3,584	109	73.4	NR	55.9	77.1	2.8	34.9	57.8	NR	NR	NR	NR	NR	NR	NR	NR	NR	High
Nakanishi, et al. (19)	Japan	12,954	923	98.5	90.6	99.3	NR	NR	NR	NR	NR	NR	NR	NR	NR	NR	76.7	NR	NR	High
Maneenil, et al. (20)	Thailand	40	40	NR	NR	52.5	10	100	55	NR	NR	NR	NR	NR	NR	NR	NR	10.5 (6.8–18.5)	NR	Moderate
Liu, et al. (21)	China	230	115	NR	NR	14.8	55.7	NR	NR	NR	NR	3.5	NR	NR	NR	NR	NR	12.7 ± 18.9	NR	High
Berger-Caron, et al. (43)	Canada	43	43	NR	NR	100	49	30	40	28	NR	5	95	NR	NR	NR	NR	23 ± 35d	21 ± 35	High
Nakwan, et al. (22)	Thailand/India/Japan/Kuwait/Pakistan/Singapore	369	369	41.5	NR	30.9	51.2	NR	17.1	8.4	NR	NR	63.4	28	25.2	18.7	8.4	5.0 (1.0–12.0)	9.0 (3.5–17.0)	High
Mat Bah, et al. (24)	Malaysia	82,915	195	57.4	NR	64.6	NR	NR	NR	NR	NR	NR	NR	NR	NR	NR	NR	NR	NR	Moderate
Lin, et al. (25)	China	203	203	NR	49.8	70	10.8	NR	23.2	15.3	NR	NR	50	NR	NR	NR	NR	NR	NR	High
Jastania, et al. (26)	Saudi Arabia	56	56	50	35.7	48.2	32.1	NR	NR	NR	NR	NR	67.9	NR	NR	NR	NR	NR	NR	Moderate
Dyess, et al. (45)	USA	224	224	NR	99	83	4.4	NR	4.5	2.2	NR	NR	2.7	NR	NR	NR	NR	NR	NR	High

HFV, high-frequency ventilation; DMV, duration of mechanical ventilation; DSO, duration of supplemental oxygen; ROB, risk of bias.

##### 3.7.3.1 Ventilation

Gentle ventilation methods that utilize an optimal combination of positive end expiratory pressure (PEEP) and mean arterial pressure (MAP), alongside moderate tidal volume and permissive hypercapnia, should be implemented. In recent years, high-frequency ventilation (HFV) has been increasingly recommended for PPHN. The proportion of included treatment studies (*n* = 212,114) that included HFV ranged from 25%–98.2%, with slight variations by region. HFV was inaccessible in LMICs.

##### 3.7.3.2 Surfactant therapy

For patients with parenchymal lung disease, such as RDS, MAS, pneumonia, etc., with primary or secondary surfactant inactivation, the concurrent PPHN emphasizes the use of pulmonary surfactant replacement therapy to recruit more alveoli and improve oxygenation (5). It is more effective in relatively mild PPHN (OI = 15–25) (67). In patients with non-parenchymal lung disease, surfactant is generally ineffective. In our studies (*n* = 212,114), the rate of PS therapy ranged from 30.7%–99%.

##### 3.7.3.3 Supporting systemic blood pressure

PPHN is often accompanied by hypotension, which requires correction with positive inotropic drugs in addition to maintenance of blood volume. While increasing blood pressure above normal levels may temporarily ameliorate right-to-left shunting through the arterial conduit, thus potentially enhancing oxygenation in the short term, it fails to alleviate pulmonary artery pressure. Moreover, all such vasoactive treatments are associated with considerable side effects and require careful administration. An extensive array of studies explores the utility of functional echocardiography, near-infrared spectroscopy, and noninvasive impedance cardiometry as supplements to standard bedside hemodynamic assessments like blood pressure and heart rate. The ideal approach for managing hemodynamic injury in neonates is determined by the underlying pathophysiology of the condition (68).

### 3.8 Specific pulmonary hypertension therapies

#### 3.8.1 Inhaled nitric oxide therapy

Inhaled nitric oxide (iNO) is the sole therapy sanctioned by the US Food and Drug Administration (FDA) for managing of PPHN in full-term or near-term neonates. This selective pulmonary vasodilator exerts minimal influence on systemic blood pressure. However, its availability is limited to a few centers within LMICs. iNO enhances systemic oxygenation in infants suffering from persistent pulmonary hypertension and has the potential to lessen the reliance on more invasive treatments (69). A randomized multicentre trial has demonstrated that treatment with high-frequency oscillatory ventilation (HFOV) combined with iNO is often more effective than treatment with either HFOV or iNO alone in cases of severe PPHN. Variation in response to treatment has been attributed in part to the specific diseases associated with PPHN (70). In our review, we found that the rate of iNO therapy for PPHN in published studies in the USA was as high as 86.1% as early as 1997 (30). Until the period of 2010–2013, it was still inconvenient in areas such as Mexico, Thailand, and Egypt (14, 37, 49). Meantime, the occurrence of residual pulmonary hypertension in term newborn infants treated with iNO for severe hypoxemic respiratory failure with associated persistent pulmonary hypertension is relatively low (71).

#### 3.8.2 Sildenafil

Sildenafil is a phosphodiesterase 5 inhibitor. Multiple studies have indicated that sildenafil is both effective and well tolerated in neonates with PPHN, particularly when inhaled nitric oxide and extracorporeal membrane oxygenation are unavailable (72–74). In our findings, sildenafil emerged as the most favored pulmonary vasodilator (77.1%), particularly in LMICs. It often serves as the initial choice of pulmonary vasodilator for treating PPHN in developing nations (75).

#### 3.8.3 Milrinone

Milrinone is classified as a phosphodiesterase 3A inhibitor, which induces pulmonary vasodilation by elevating levels of cyclic adenosine monophosphate (cAMP). Serving as both an inotropic agent and a vasodilator for both systemic and pulmonary circulation (76), it is regarded as the preferred treatment option for PPHN accompanied by left ventricular dysfunction. Additionally, milrinone is utilized for patients who do not respond to iNO and serves as a secondary option to sildenafil. Multiple studies indicate that the use of milrinone enhances oxygenation in cases of PPHN (77–81). This review included the use of milrinone in 6 trials (18, 20, 22, 25, 54, 66).

#### 3.8.4 Bosentan

Bosentan was the subject of a randomized, double-blind, placebo-controlled, prospective investigation conducted in Egypt aimed at evaluating its efficacy in treating PPHN (66). The study concluded that bosentan might serve as a beneficial adjuvant therapy in managing PPHN (82), with comparable findings reported in additional trials. However, bosentan has been linked to several adverse effects, including abnormal liver function, anemia, and edema.

#### 3.8.5 Prostaglandins

Prostaglandin E1, known for its pulmonary vasodilatory effects, is administered in both intravenous and nebulized forms for treating PPHN. A total of seven studies of prostaglandin treatment in PPHN were included in our review (14, 17, 18, 22, 25, 54, 66, 83). They had a role in improving oxygenation, but their safety and efficacy need to be further evaluation.

#### 3.8.6 ECMO

The efficacy of ECMO is certain in severe PPHN with or without concomitant heart failure. With the widespread use of iNO and high frequency ventilation (HFV), there has been a relative decline in the number of cases requiring ECMO. The criteria for ECMO application can vary among different medical centers. According to the Guidelines for Neonatal Respiratory Failure from the Extracorporeal Life Support Organization (ELSO), the indications for ECMO include: (1) insufficient tissue oxygenation despite maximal therapeutic efforts; (2) severe hypoxic respiratory failure accompanied by acute decompensation (PaO2 < 40 mmHg); (3) sustained elevation of the oxygenation index without improvement; (4) significant pulmonary hypertension coupled with signs of right and/or left ventricular dysfunction. Contraindications encompass: (1) lethal chromosomal disorders (such as trisomy 13, 18, but not 21) or other lethal anomalies; (2) severe brain injury; (3) uncontrollable hemorrhage; (4) substantial intraventricular hemorrhage; (5) inadequate vessel size for cannulation. A systematic review involving 1,814 neonates indicated that ECMO can be effectively applied in neonates suffering from PPHN who have not responded to supportive cardiorespiratory care and conventional treatments, achieving a neonatal survival rate of 67.1% (84).

## 4 Discussion

This paper examines the current literature on PPHN to ascertain its worldwide prevalence, mortality rates, causes, risk factors, diagnostic methods, and treatment options. Our findings indicate that PPHN presents significant morbidity and mortality challenges in low-resource environments. Neonatal infections emerged as the primary contributor to PPHN and the leading cause of PPHN-related fatalities. Risk indicators for PPHN can be categorized into maternal, fetal, and obstetric factors. We outline the prevalent causes and existing treatments for PPHN and conclude by recommending a straightforward, practical algorithm for prompt diagnosis and management.

### 4.1 The global prevalence of PPHN

The global prevalence of PPHN The findings regarding the prevalence of PPHN indicate a significant variability across the 43 studies analyzed, influenced by multiple factors. Early investigations conducted in central Thailand identified an incidence of PPHN that fluctuated between 0.38 and 0.99 per 1,000 live births (85). In contrast, research from southern Thailand revealed a higher incidence of 2.8 per 1,000 live births (17). Prior to the extensive application of iNO therapy, data from a multicenter study in the United States reported a PPHN prevalence of 1.9 per 1,000 live births (from a cohort of 71,558 infants), with marked variation noted among different centers, ranging from 0.43–6.82 per 1,000 live births) (33). A separate investigation focusing on late preterm and term infants in California found an incidence of PPHN at 1.8 per 1,000 live births (0.18%).Notably, late preterm infants (gestational age between 34 and 36 weeks) had the highest incidence at 5.4 per 1,000 live births, whereas term infants presented a lower incidence of 1.6 per 1,000 live births (40). A retrospective review of medical records from several centers across six Asian nations (Japan, Kuwait, India, Pakistan, Singapore, and Thailand) conducted between January 1, 2014, and December 31, 2016, demonstrated that the incidence of PPHN varied from 1.2–4.6 per 1,000 live births (22). Additionally, a decade-long multicenter study involving extremely preterm infants (*n* = 12,954 cases) in Japan reported a prevalence of PPHN at 8.1% (95% CI 7.7%–8.6%), which showed an upward trend annually, and a notably higher prevalence with decreasing gestational age: 18.5% (range, 15.2%–22.4%) for those born at 22 weeks, vs. 4.4% (range, 3.8%–5.2%) for infants born at 27 weeks (19). The prevalence of PPHN is influenced by geographical locations, population characteristics, and gestational age. As illustrated by the aforementioned data, countries with the highest and lowest prevalence rates of PPHN suggest that low- and middle-income countries (LMICs), particularly in Africa and Asia, are disproportionately affected compared to high-income countries (HICs) in the Americas and Europe. Additionally, several advancements in neonatal care, including the creation of NICUs, the induction of fetal lung development through antenatal steroid administration, refinement of neonatal resuscitation protocols, the use of exogenous surfactant immature lungs, and the development of advanced respiratory support techniques like continuous positive airway pressure (CPAP), HFV, are often inadequate or lacking in LMICs. Moreover, the significant variability in prevalence rates can be attributed to differences in research methodologies, which report varying figures for the clinical diagnosis of PPHN. The diagnostic criteria for PPHN have shown inconsistencies, potentially leading to both underestimation and overestimation of its prevalence. Furthermore, study context might contribute to this variability; it was anticipated that single-center studies would report lower PPHN prevalence rates compared to multicenter studies. However, data revealed that PPHN prevalence in single-center investigations ranged from 0.1%–5.7%, while multicenter studies reported lower rates of 0.12%–8.1%. This discrepancy could be due to single-center studies being conducted in specialized neonatal care facilities, whereas multicenter studies were carried out across a broader range of institutions (see Table 2). Additionally, the prevalence variations across the 43 included studies may reflect the differing gestational ages of the newborns involved: from 8.3%–100% in premature infants (7, 19, 21–26, 32, 35, 38–40, 42, 47, 48, 51, 54, 58, 66, 86) compared to exclusively extremely preterm infants (19). Collectively, these results suggest that the incidence of PPHN diminishes with advancing gestational age, which is attributed to the functional and structural immaturity of the lungs in premature neonates.

### 4.2 Etiologies of PPHN and PPHN-related in-hospital mortality

Between 1993 and 2023, there has been no noteworthy reduction in mortality rates associated with PPHN. The fact that neonatal infections remain the primary cause of PPHN and contribute significantly to in-hospital fatalities suggests that, despite advancements in the management of PPHN over the last thirty years, additional therapeutic developments are still necessary. These initiatives should be especially focused on the Americas and Asia, where neonates experience varying impacts from neonatal infections. As expected, preventable neonatal infections remain a major global health problem, particularly in Asia and Africa. A preponderance of congenital diaphragmatic hernia and/or pulmonary dysplasia in the Americas may reflect a high local prevalence of congenital anomalies. More research is required to explore the role of environmental, genetic, and other factors alongside early prevention strategies.

### 4.3 Predictors or risk factors associated with PPHN

Factors that may predict the occurrence of PPHN include cesarean section, which is widely acknowledged as a contributing risk factor for this condition. A study conducted by Winovitch indicated that the rate of PPHN among those undergoing elective cesarean delivery was 6.9 per 1,000 births (61). Various research efforts have shown a greater prevalence of PPHN in infants born via cesarean section (CS) compared to those delivered vaginally (VD) (35, 55, 61, 87, 88). Specifically, the occurrence of PPHN was found to be around 0.37% in neonates delivered by CS, nearly five times higher than in those delivered vaginally (88). Elective cesarean sections, in particular, disrupt the physiological processes, such as the release of catecholamines and glucocorticoids that promote pulmonary fluid absorption, surfactant production, and pulmonary vasodilation, which are critical for the newborn's normal cardiorespiratory adjustment during labor (87, 89). Furthermore, fetal distress frequently leads to cesarean deliveries, which is another prevalent risk factor for PPHN. Consequently, the elevated rate of PPHN associated with CS may primarily stem from the activation of latent fetal factors rather than from a direct causal link with CS or the absence of VD. Notably, the incidence of PPHN in infants delivered through elective cesarean procedures before labor begins is significantly higher than in those born vaginally (OR = 4.9, 95% CI 1.7–14.0), indicating that cesarean delivery itself constitutes a risk factor for PPHN (60). CS has been linked to respiratory distress syndrome (RDS), which poses a higher mortality risk from PPHN in preterm infants compared to those born at term or post-term (51). Therefore, it is critical for obstetricians to conduct a comprehensive evaluation during CS procedures, particularly in cases of preterm labor. Meconium aspiration syndrome (MAS) is recognized as the leading contributor to PPHN. The presence of meconium interferes with pulmonary surfactants, triggering lung inflammation and causing alveolar hypoxia, which results in pulmonary vasoconstriction. Furthermore, meconium lodged in the airways can cause obstruction, gas trapping, and increased pulmonary vascular resistance. Nevertheless, the relationship between PPHN and meconium aspiration remains ambiguous; it is uncertain whether PPHN emerges directly from the aspiration or serves as an indirect indicator of *in utero* stress factors, such as hypoxia or infection. Additionally, a multicenter retrospective study of preterm infants indicated that clinical chorioamnionitis and premature rupture of membranes were correlated with PPHN (19). Inflammatory mediators leading to functional or structural pulmonary deficits, alongside severe infections that may compromise circulation or induce shock, could contribute to the emergence of PPHN, potentially accounting for the rising prevalence of this condition, particularly among preterm births.

Regarding the fetal risk factors related to delayed pulmonary biomechanics and vascular development, elevated levels of serum androgens in males compared to females contribute significantly (90, 91). Testosterone delays fetal lung maturation by regulating growth factors, and fetal androgens also inhibit fetal lung surfactant secretion. Meanwhile, estrogen promotes the synthesis of surfactant components, the growth of type II lung cells, and an overall increase in fetal lung surfactant secretion (92, 93). RDS is frequently a contributing factor to PPHN. Consequently, preterm infants face a greater risk of developing PPHN than their term counterparts. The insufficient synthesis and release of lung surfactant along with a limited number of respiratory units in preterm infants make them more susceptible to atelectasis and, ultimately, hypoxia associated with RDS. Factors such as poor lung compliance, reduced tidal volumes, increased physiological dead space, and inadequate alveolar ventilation lead to hypercapnia. The interplay of hypercapnia, hypoxia, and acidosis results in pulmonary vasoconstriction and enhanced right-to-left shunting through the patent foramen ovale and arterial ducts, as well as within the lungs, all of which exacerbate the risk of PPHN. GDM and PGDM result in intrauterine exposure to maternal hyperglycemia, which increases the transplacental transfer of glucose to the fetus, resulting in fetal hyperglycemia. This fetal hyperglycemia, in turn, inhibits the synthesis of fetal lung surfactant. Adverse outcomes are more prevalent in patients with inadequate blood glucose control, such as RDS (*χ*² = 13.373, *P* < 0.01). In addition, elevated fasting plasma glucose (FPG) has been deemed an independent risk factor for preterm birth, with an odds ratio (OR) of 1.460 (*P* < 0.001) (94). Simultaneously, there is a significant correlation between maternal obesity and advanced maternal age with an increased likelihood of experiencing obstetric complications, including maternal diabetes/GDM, pregnancy-related hypertensive disorders, and antepartum hemorrhage, all of which can predispose PPHN. All of these increase the probability of a cesarean section. Fetal distress and APGAR scores at birth often have interlocking or cascading mechanisms in predicting PPHN. Moreover, both hypoxia and acidosis are unequivocally powerful pulmonary vasoconstrictors, blocking changes in the cardiopulmonary circulation in normal newborns after birth, therefore, any factors leading to intrauterine hypoxia may play a role in the onset of PPHN.

The topic of using antidepressants during pregnancy remains contentious. Selective serotonin reuptake inhibitors (SSRIs) have been linked to a higher incidence of severe cardiac malformations as well as persistent pulmonary hypertension of the newborn (PPHN). Nevertheless, the majority of neurodevelopmental studies tracking follow-up have not uncovered significant cognitive impairments, observing only minor transient gross motor delays, slight language issues, and potential behavioral modifications. The dangers associated with halting treatment seem to surpass those tied to continuing it, as serious maternal depression could adversely affect a child's development. If deemed necessary, it is advisable to maintain treatment throughout pregnancy at the minimum effective dosage (95, 96).

It remains uncertain whether some of these risk factors are definitively a direct cause of persistent pulmonary hypertension in newborns or if they are merely co-contributors. Genetic variations have been identified in individuals with PPHN. Research within a single-center Chinese cohort has identified CPS1, NOTCH3, and SMAD9 as genetic risk factors for late preterm and term PPHN (21). These discoveries contribute further genetic support to the understanding of PPHN's pathogenesis and provide new perspectives on potential therapeutic strategies for the condition. Given the elevated risk of mortality and poor outcomes for survivors of PPHN, healthcare providers must be particularly attentive to the heightened need for monitoring and intervention in pregnant women or newborns presenting these risk factors. In addition, the influence of maternal factors, (e.g., maternal diabetes, maternal obesity, hypertensive disorders of pregnancy), on offspring warrant further exploration. In the future, in addition to databases of children with PPHN, more high-quality cohorts and databases of targeted therapeutic agents used could be established, especially in low- and middle-income settings, with closer and longer follow-up to assess the long-term impact.

The application of inhaled nitric oxide (iNO) for treating pulmonary hypertension (PH) in newborns delivered at less than 34 weeks of gestation has been extensively reviewed. Meta-analyses from randomized controlled trials focusing on the use of iNO for preventing bronchopulmonary dysplasia (BPD) have led groups from the National Institutes of Health (NIH) and the American Academy of Pediatrics (AAP) to determine that there is insufficient evidence to support the routine implementation of iNO in preterm infants. Nonetheless, existing research indicates that administering iNO can enhance oxygen levels in these infants, and the inclination to employ iNO is on the rise. The NIH consensus recommends that healthcare providers must evaluate the appropriateness of iNO treatment for preterm infants suffering from PH. Additionally, the American Heart Association, American Thoracic Society, and the Pediatric Pulmonary Hypertension Collaborative Network endorse the application of iNO in cases of persistent pulmonary hypertension of the newborn (PPHN) when standard respiratory and circulatory interventions fail to yield adequate results. It's important to note that both iNO and ECMO are not universally accessible and pose significant challenges in low- and middle-income countries (LMICs). Moreover, around 40% of neonates diagnosed with PPHN do not respond to iNO therapy (8). ECMO represents a resource-heavy treatment modality that necessitates a collaborative approach from a team of skilled healthcare professionals with specialized training in the setup, ongoing care, and cessation of ECMO for critically ill PPHN patients facing diverse causes and associated health complications. The principal risks linked to ECMO include bleeding, clot formation, and infections related to catheters. Comprehensive preparation, strategic resource distribution, and rigorous training of personnel to implement complex treatment strategies while following stringent infection control protocols are vital aspects of any ECMO operational strategy. There is a pressing need for additional prospective multi-institutional research to broaden the existing registry data.

Consequently, further interventions are under investigation. Recent years have seen significant progress in our comprehension of the physiopathology associated with PPHN, encompassing elements such as phosphodiesterase, endothelin receptors, and the supplementation of exogenous prostaglandins. These medications can be utilized individually or in conjunction with one another or with iNO. Sildenafil has undergone the most comprehensive research. A systematic review and network meta-analysis exploring various PPHN treatment comparisons indicated that a median concentration of 10–20 parts per million (ppm) of iNO (MNO), in combination with sildenafil given orally at a dose of 1–3 mg/kg every 6–8 h (OSID), showed the highest efficacy (OR = 27.53, 95% CI 2.36–321.75). In situations where iNO is unavailable, OSID paired with intravenous milrinone also demonstrated commendable efficacy (OR = 25.13, 95% CI 1.67–377.78) (97). PPHN is a complex and variable disease, and it is challenging for clinicians to make the right choice of individual drugs based on good evidence in routine practice. Meanwhile, most vasodilator drugs are still used outside the prescription instructions in neonates. Their clinical efficacy and potential and long-term adverse reactions are still being monitored and followed-up. At the same time, the treatment of PPHN is complex, expensive and limited in resource-limited regions. Alternative or cheaper treatments are being sought. Moreover, because the signaling pathways that control pulmonary vascular tone are intricate and highly interconnected, addressing only one particular pathway may not fully rectify vascular anomalies and could potentially upset the equilibrium between vasodilator and vasoconstrictor production. Each of the therapies mentioned may have significance, and a combined approach utilizing multiple treatments could be beneficial in certain severe cases.

Employing a combination of therapies could enhance outcomes in certain severe instances. A significant drawback of the present research is the variability among all studies included, which complicates the meta-analysis. Specifically, conducting a meta-analysis utilizing combined prevalence, mortality rates for PPHN, and risk assessments is unsuitable due to the diverse methodological approaches present in the studies examined. Nonetheless, this scoping review acts as a modern reference document that has been globally referenced for PPHN throughout the last three decades. It lays the foundation for systematic reviews and meta-analyses pertaining to epidemiological research on PPHN.

## 5 Conclusions

The prevalence of PPHN is particularly concerning in preterm infants and those with respiratory issues. Major contributors to PPHN include neonatal infections and meconium aspiration syndrome (MAS). Various identified risk factors (such as gestational age, birth weight, maternal smoking habits, diabetes mellitus-both pregestational and gestational, maternal obesity, prenatal infections, fetal distress, APGAR score, and meconium-stained amniotic fluid) are considered modifiable. Therefore, we suggest implementing changes in maternal lifestyle, ensuring appropriate antenatal, intrapartum, and postnatal care to mitigate these risks and improve the management strategies for PPHN. Additionally, the pressing demand for enhanced treatment methods and innovations aimed at PPHN, ideally supported in settings with limited resources, cannot be overstated as a crucial step to reduce the high mortality rates associated with PPHN.
