# Supplementary material for: Global, regional and national trends in the burden of persistent pulmonary hypertension of the newborn and essentials of its management from 1993 to 2023: a scoping review
Source: Front Pediatr. 2025 Jun 4;13:1502385. doi: 10.3389/fped.2025.1502385 (PMC12174464; doi:10.3389/fped.2025.1502385)
Supplement: Supplementary file 1 [file Table1.docx]

Supplementary Material

**Contents of Supplementary File:**

**Supplementary File S1. PRISMA-ScR Checklist for this scoping review.**

**Supplementary File S2. Search strategies.**

**Supplementary File S3. Search strategies(simplified version).**

# Supplementary File S4. The NOS to rate the risk of bias in cohort and case-control studies

# Supplementary File S5. The AHRQ to rate the risk of bias in cross-sectional studies.

# Supplementary File S6. The Jadad to rate the risk of bias in RCT studies

# Supplementary File S1. Preferred Reporting Items for Systematic reviews and Meta-Analyses extension for Scoping Reviews (PRISMA-ScR) Checklist.

| **SECTION** | **ITEM** | **PRISMA-ScR CHECKLIST ITEM** | **REPORTED ON PAGE #** |
| --- | --- | --- | --- |
| **TITLE** | | | |
| Title | 1 | Identify the report as a scoping review. | 1 |
| **ABSTRACT** | | | |
| Structured summary | 2 | Provide a structured summary that includes (as applicable): background, objectives, eligibility criteria, sources of evidence, charting methods, results, and conclusions that relate to the review questions and objectives. | 1 and 2 |
| **INTRODUCTION** | | | |
| Rationale | 3 | Describe the rationale for the review in the context of what is already known. Explain why the review questions/objectives lend themselves to a scoping review approach. | 2 and 3 |
| Objectives | 4 | Provide an explicit statement of the questions and objectives being addressed with reference to their key elements (e.g., population or participants, concepts, and context) or other relevant key elements used to conceptualize the review questions and/or objectives. | 3 |
| **METHODS** | | | |
| Protocol and registration | 5 | Indicate whether a review protocol exists; state if and where it can be accessed (e.g., a Web address); and if available, provide registration information, including the registration number. | None |
| Eligibility criteria | 6 | Specify characteristics of the sources of evidence used as eligibility criteria (e.g., years considered, language, and publication status), and provide a rationale. | 3 and 4 |
| Information sources* | 7 | Describe all information sources in the search (e.g., databases with dates of coverage and contact with authors to identify additional sources), as well as the date the most recent search was executed. | 3 and 4 |
| Search | 8 | Present the full electronic search strategy for at least 1 database, including any limits used, such that it could be repeated. | 3 and 4 |
| Selection of sources of evidence† | 9 | State the process for selecting sources of evidence (i.e., screening and eligibility) included in the scoping review. | 4 |
| Data charting process‡ | 10 | Describe the methods of charting data from the included sources of evidence (e.g., calibrated forms or forms that have been tested by the team before their use, and whether data charting was done independently or in duplicate) and any processes for obtaining and confirming data from investigators. | 4 |
| Data items | 11 | List and define all variables for which data were sought and any assumptions and simplifications made. | 3 and 4 |
| Critical appraisal of individual sources of evidence§ | 12 | If done, provide a rationale for conducting a critical appraisal of included sources of evidence; describe the methods used and how this information was used in any data synthesis (if appropriate). | None |
| Synthesis of results | 13 | Describe the methods of handling and summarizing the data that were charted. | 4 |
| **RESULTS** | | | |
| Selection of sources of evidence | 14 | Give numbers of sources of evidence screened, assessed for eligibility, and included in the review, with reasons for exclusions at each stage, ideally using a flow diagram. | 4 |
| Characteristics of sources of evidence | 15 | For each source of evidence, present characteristics for which data were charted and provide the citations. | 4 |
| Critical appraisal within sources of evidence | 16 | If done, present data on critical appraisal of included sources of evidence (see item 12). | None |
| Results of individual sources of evidence | 17 | For each included source of evidence, present the relevant data that were charted that relate to the review questions and objectives. | 5 to 10 |
| Synthesis of results | 18 | Summarize and/or present the charting results as they relate to the review questions and objectives. | 5 to 10 |
| **DISCUSSION** | | | |
| Summary of evidence | 19 | Summarize the main results (including an overview of concepts, themes, and types of evidence available), link to the review questions and objectives, and consider the relevance to key groups. | 10 to 13 |
| Limitations | 20 | Discuss the limitations of the scoping review process. | 13 |
| Conclusions | 21 | Provide a general interpretation of the results with respect to the review questions and objectives, as well as potential implications and/or next steps. | 13 |
| **FUNDING** | | | |
| Funding | 22 | Describe sources of funding for the included sources of evidence, as well as sources of funding for the scoping review. Describe the role of the funders of the scoping review. | 14 |

# Supplementary File S2. Search strategies

**PubMed**

| PubMed | | |
| --- | --- | --- |
| Search number | Query | Results |
| 1 | "Infant, Newborn"[Mesh] | [692,988](https://pubmed.ncbi.nlm.nih.gov/?term=%22Infant%2C+Newborn%22%5BMesh%5D&sort=relevance) |
| 2 | ((Newborn[Title/Abstract]) OR (Infant[Title/Abstract])) OR (neonat*[Title/Abstract]) | 640,665 |
| 3 | "Persistent Fetal Circulation Syndrome"[Mesh] | 1,344 |
| 4 | (((Persistent Fetal Circulation Syndrome[Title/Abstract]) OR (Persistent Pulmonary Hypertension of Newborn[Title/Abstract])) OR (Persistent Pulmonary Hypertension of the Newborn[Title/Abstract])) OR (Persistent Fetal Circulation[Title/Abstract]) | 1,329 |
| 5 | "Prevalence"[Mesh] | 358,233 |
| 6 | "Incidence"[Mesh] | 310,967 |
| 7 | "Mortality"[Mesh] | 431,715 |
| 8 | (((((((((((Profile[Title/Abstract]) OR (Spectrum[Title/Abstract])) OR (Fatality[Title/Abstract])) OR (Neonatal Death[Title/Abstract])) OR (Risk factors[Title/Abstract])) OR (Predictors[Title/Abstract])) OR (Determinants[Title/Abstract])) OR (Etiologies[Title/Abstract])) OR (Aetiologies[Title/Abstract])) OR (Causes[Title/Abstract])) OR (Management[Title/Abstract])) OR (Treatment[Title/Abstract]) | 8,682,309 |
| 9 | ("Infant, Newborn"[Mesh]) OR (((Newborn[Title/Abstract]) OR (Infant[Title/Abstract])) OR (neonat*[Title/Abstract])) | 1,035,475 |
| 10 | ("Persistent Fetal Circulation Syndrome"[Mesh]) OR ((((Persistent Fetal Circulation Syndrome[Title/Abstract]) OR (Persistent Pulmonary Hypertension of Newborn[Title/Abstract])) OR (Persistent Pulmonary Hypertension of the Newborn[Title/Abstract])) OR (Persistent Fetal Circulation[Title/Abstract])) | 2,077 |
| 11 | ((("Prevalence"[Mesh]) OR ("Incidence"[Mesh])) OR ("Mortality"[Mesh])) OR ((((((((((((Profile[Title/Abstract]) OR (Spectrum[Title/Abstract])) OR (Fatality[Title/Abstract])) OR (Neonatal Death[Title/Abstract])) OR (Risk factors[Title/Abstract])) OR (Predictors[Title/Abstract])) OR (Determinants[Title/Abstract])) OR (Etiologies[Title/Abstract])) OR (Aetiologies[Title/Abstract])) OR (Causes[Title/Abstract])) OR (Management[Title/Abstract])) OR (Treatment[Title/Abstract])) | 9,284,715 |
| 12 | ((("Infant, Newborn"[Mesh]) OR (((Newborn[Title/Abstract]) OR (Infant[Title/Abstract])) OR (neonat*[Title/Abstract]))) AND (("Persistent Fetal Circulation Syndrome"[Mesh]) OR ((((Persistent Fetal Circulation Syndrome[Title/Abstract]) OR (Persistent Pulmonary Hypertension of Newborn[Title/Abstract])) OR (Persistent Pulmonary Hypertension of the Newborn[Title/Abstract])) OR (Persistent Fetal Circulation[Title/Abstract])))) AND (((("Prevalence"[Mesh]) OR ("Incidence"[Mesh])) OR ("Mortality"[Mesh])) OR ((((((((((((Profile[Title/Abstract]) OR (Spectrum[Title/Abstract])) OR (Fatality[Title/Abstract])) OR (Neonatal Death[Title/Abstract])) OR (Risk factors[Title/Abstract])) OR (Predictors[Title/Abstract])) OR (Determinants[Title/Abstract])) OR (Etiologies[Title/Abstract])) OR (Aetiologies[Title/Abstract])) OR (Causes[Title/Abstract])) OR (Management[Title/Abstract])) OR (Treatment[Title/Abstract]))) | 1,127 |
| 13 | (("infant, newborn"[MeSH Terms] OR ("Newborn"[Title/Abstract] OR "Infant"[Title/Abstract] OR "neonat*"[Title/Abstract])) AND ("Persistent Fetal Circulation Syndrome"[MeSH Terms] OR ("Persistent Fetal Circulation Syndrome"[Title/Abstract] OR "persistent pulmonary hypertension of newborn"[Title/Abstract] OR "persistent pulmonary hypertension of the newborn"[Title/Abstract] OR "persistent fetal circulation"[Title/Abstract])) AND ("Prevalence"[MeSH Terms] OR "Incidence"[MeSH Terms] OR "Mortality"[MeSH Terms] OR ("Profile"[Title/Abstract] OR "Spectrum"[Title/Abstract] OR "Fatality"[Title/Abstract] OR "neonatal death"[Title/Abstract] OR "risk factors"[Title/Abstract] OR "Predictors"[Title/Abstract] OR "Determinants"[Title/Abstract] OR "Etiologies"[Title/Abstract] OR "Aetiologies"[Title/Abstract] OR "Causes"[Title/Abstract] OR "Management"[Title/Abstract] OR "Treatment"[Title/Abstract]))) AND (1993/1/1:2023/12/31[pdat]) | 956 |

**Cochrane Library**

| Cochrane library | | |
| --- | --- | --- |
| ID | Search | Hits |
| #1 | MeSH descriptor: [Persistent Fetal Circulation Syndrome] explode all trees | 84 |
| #2 | (persistent pulmonary hypertension of newborn):ti,ab,kw OR (persistent fetal circulation syndrome):ti,ab,kw OR (persistent pulmonary hypertension of the newborn):ti,ab,kw OR (persistent fetal circulation):ti,ab,kw (Word variations have been searched) | 266 |
| #3 | MeSH descriptor: [Infant, Newborn] explode all trees | 24173 |
| #4 | (Newborn):ti,ab,kw OR (Infant):ti,ab,kw OR (neonat*):ti,ab,kw (Word variations have been searched) | 91864 |
| #5 | MeSH descriptor: [Prevalence] explode all trees | 7419 |
| #6 | MeSH descriptor: [Incidence] explode all trees | 14390 |
| #7 | MeSH descriptor: [Mortality] explode all trees | 18908 |
| #8 | (Prevalence):ti,ab,kw OR (Incidence):ti,ab,kw OR (Mortality):ti,ab,kw OR (Profile):ti,ab,kw OR (Spectrum):ti,ab,kw (Word variations have been searched) | 407726 |
| #9 | (Fatality):ti,ab,kw OR (Neonatal Death):ti,ab,kw OR (Risk factors):ti,ab,kw OR (Predictors):ti,ab,kw OR (Determinants):ti,ab,kw (Word variations have been searched) | 445939 |
| #10 | (Etiologies):ti,ab,kw OR (Aetiologies):ti,ab,kw OR (Causes):ti,ab,kw OR (Management):ti,ab,kw OR (Treatment):ti,ab,kw (Word variations have been searched) | 1178584 |
| #11 | #1 OR #2 | 266 |
| #12 | #3 OR #4 | 91864 |
| #13 | #5 OR #6 OR #7 OR #8 OR #9 OR #10 | 1433805 |
| #14 | #11 AND #12 AND #13 | 233 |
| #15 | #11 AND #12 AND #13 with Cochrane Library publication date Between Jan 1993 and Dec 2023 | 226 |

**Supplementary File S3. Search strategies (simplified version).**

| Search number | Search terms |
| --- | --- |
| #1 | Persistent Fetal Circulation Syndrome [MeSH] |
| #2 | persistent pulmonary hypertension of newborn[TIAB] OR persistent fetal circulation syndrome[TIAB] OR persistent pulmonary hypertension of the newborn[TIAB] OR persistent fetal circulation[TIAB] |
| #3 | Infant, Newborn [MeSH] |
| #4 | Newborn[TIAB] OR Infant[TIAB] OR neonat*[TIAB] |
| #5 | Prevalence [MeSH] |
| #6 | Incidence [MeSH] |
| #7 | Mortality [MeSH] |
| #8 | Prevalence[TIAB] OR Incidence[TIAB] OR Mortality[TIAB] OR Profile[TIAB] OR Spectrum[TIAB] |
| #9 | Fatality[TIAB] OR Neonatal Death[TIAB] OR Risk factors[TIAB] OR Predictors[TIAB] OR Determinants[TIAB] |
| #10 | Etiologies[TIAB] OR Aetiologies[TIAB] OR Causes[TIAB] OR Management[TIAB] OR Treatment[TIAB] |
| #11 | #1 OR #2 |
| #12 | #3 OR #4 |
| #13 | #5 OR #6 OR #7 OR #8 OR #9 OR #10 |
| #14 | #11 AND #12 AND #13 |
| #15 | Limits: publication date Between Jan 1993 and Dec 2023 with no language restriction |

# Supplementary File S4. The NOS to rate the risk of bias in cohort and case-control studies

| **References** | **Selection** | | | | **Comparability** |  | **Outcomes** | | | **Total (max score: 9)** |
| --- | --- | --- | --- | --- | --- | --- | --- | --- | --- | --- |
|  | **Representative-**  **ness of the exposed cohort** | **Selection of the non-exposed cohort** | **Ascertainment of exposure to implants** | **Demonstration**  **that outcome of interest was not present at start of study** | **Comparability**  **of cohorts on the basis of the design or analysis** | **Comparability**  **of cohorts on the basis of the measurement** | **Assessment of outcome** | **Was follow up long enough for outcomes to occur** | **Adequacy of follow up of cohorts** |  |
| Rosenberg, 1997(30) | **★** | **★** | **★** | **★** | **★** | **★** | **★** | **★** | **NO** | 8 |
| Favilli, 1998(46) | **★** | **★** | **★** | **★** | NO | **★** | **★** | **NO** | **NO** | 6 |
| Mok, 1999(47) | **★** | **★** | **★** | **★** | **★** | **NO** | **★** | **NO** | **NO** | 6 |
| Walsh-Sukys, 2000(34) | **★** | **★** | **★** | **★** | **★** | **★** | **★** | **NO** | NO | 8 |
| Torielli, 2001(73) | **★** | **★** | **★** | **★** | **★** | **★** | **★** | **★** | NO | 8 |
| Levine, 2001(88) | **★** | **★** | **★** | **★** | **★** | **★** | **★** | **NO** | **NO** | 7 |
| Su, 2002(13) | **★** | **★** | **★** | **★** | **★** | **★** | **★** | **NO** | **NO** | 7 |
| Lipkin, 2002(11) | **★** | **★** | **★** | **★** | **★** | **★** | **★** | **★** | **NO** | 8 |
| Clark, 2003(35) | **★** | **★** | **★** | **★** | **★** | **NO** | **★** | **★** | **NO** | 7 |
| Hwang, 2004(56) | **★** | **★** | **★** | **★** | **★** | **NO** | **★** | **NO** | **NO** | 6 |
| Pierce, 2004(48) | **★** | **★** | **★** | **★** | **★** | **★** | **★** | **NO** | **NO** | 7 |
| Kumar, 2007(61) | **★** | **★** | **★** | **★** | **★** | NO | **★** | **NO** | **NO** | 6 |
| Hernández-Díaz, 2007(36) | **★** | **★** | **★** | **★** | **★** | **★** | **★** | **★** | **★** | 9 |
| Eriksen, 2009(57) | **★** | **★** | **★** | **★** | **★** | **★** | **★** | **★** | **NO** | 8 |
| Peterson, 2009(37) | **★** | **★** | **★** | **★** | **★** | **NO** | **★** | **★** | **★** | 8 |
| Ortiz, 2010(38) | **★** | **★** | **★** | **★** | NO | NO | **★** | **★** | **NO** | 6 |
| Berti, 2010(49) | **★** | **★** | **★** | **★** | **★** | **★** | **★** | **★** | **★** | 9 |
| Roofthooft, 2011(51) | **★** | **★** | **★** | **★** | ★ | NO | **★** | **★** | **NO** | 7 |
| Nakwan, 2011(14) | **★** | **★** | **★** | **★** | **★** | **NO** | **★** | **★** | **★** | 7 |
| Byers, 2012(39) | **★** | **★** | **★** | **★** | **★** | **★** | **★** | **★** | **★** | 9 |
| Rocha, 2012(52) | **★** | **★** | **★** | **★** | **★** | NO | **★** | **★** | **★** | 7 |
| Abdel Mohsen, 2013(53) | **★** | **★** | **★** | **★** | **★** | **★** | **★** | **★** | **NO** | 8 |
| Razzaq, 2013(29) | **★** | **★** | **★** | **★** | **★** | **NO** | **★** | **★** | **★** | 7 |
| Janjindamai, 2013(16) | **★** | **★** | **★** | **★** | **★** | **★** | **★** | **★** | **NO** | 8 |
| Yao, 2013(55) | **★** | **★** | **★** | **★** | **★** | **NO** | **★** | **★** | **NO** | 7 |
| Malowitz, 2015(40) | **★** | **★** | **★** | **★** | **★** | **★** | **★** | **★** | **★** | 9 |
| Nakwan, 2016(17) | **★** | **★** | **★** | **★** | **★** | NO | **★** | NO | NO | 6 |
| Steurer, 2017(41) | **★** | **★** | **★** | **★** | **★** | **★** | **★** | **★** | **★** | 9 |
| Kamolvisit, 2018(18) | **★** | **★** | **★** | **★** | **★** | **NO** | **★** | **★** | **NO** | 7 |
| Nakanishi, 2018(19) | **★** | **★** | **★** | **★** | **★** | **★** | **★** | **★** | NO | 8 |
| Maneenil, 2018(20) | **★** | **★** | **★** | **★** | **★** | NO | **★** | **★** | **NO** | 6 |
| Steurer, 2019(42) | **★** | **★** | **★** | **★** | **★** | **★** | **★** | **★** | **★** | 9 |
| Liu, 2019(21) | **★** | **★** | **★** | **★** | **★** | **★** | **★** | **★** | **★** | 9 |
| Berger-Caron, 2019(43) | **★** | **★** | **★** | **★** | **★** | **NO** | **★** | **★** | **★** | 8 |
| Nakwan, 2020(22) | **★** | **★** | **★** | **★** | **★** | NO | **★** | **★** | **NO** | 7 |
| Arshad, 2021(23) | **★** | **★** | **★** | **★** | NO | NO | **★** | **★** | **NO** | 6 |
| Aleem, 2021(44) | **★** | **★** | **★** | **★** | **★** | **★** | **★** | **★** | **NO** | 8 |
| Lin, 2022(25) | **★** | **★** | **★** | **★** | **★** | NO | **★** | **★** | **NO** | 7 |
| Qian, 2022(27) | **★** | **★** | **★** | **★** | **★** | **★** | **★** | **★** | **NO** | 8 |
| Kamran, 2022(28) | **★** | **★** | **★** | **★** | **NO** | NO | **★** | **★** | **NO** | 6 |
| Dyess,2023(45) | **★** | **★** | **★** | **★** | **★** | NO | **★** | **★** | **★** | 8 |
| Wei, 2023(85) | **★** | **★** | **★** | **★** | **★** | NO | **★** | **★** | **NO** | 7 |

# Supplementary File S5. The AHRQ to rate the risk of bias in cross-sectional studies.

| **References** | **1. Define the source of information (survey, record review)** | **2. List inclusion and exclusion criteria for exposed and unexposed subjects (cases and controls) or refer to previous publications** | **3. Indicate time period used for identifying patients** | **4. Indicate whether or not subjects were consecutive if not population-based** | **5. Indicate if evaluators of subjective components of study were masked to other aspects of the status of the participants** | **6. Describe any assessments undertaken for quality assurance purposes (e.g., test/retest of primary outcome measurements** | **7.Explain any patient exclusions from analysis** | **8. Describe how confounding was assessed and/or controlled.** | **9. If applicable, explain how missing data were handled in the analysis** | **10.Summarize patient response rates and completeness of data collection** | **11.Clarify what follow-up, if any, was expected and the percentage of patients for which incomplete data or follow-up was obtained** | **Total (max score: 11)** |
| --- | --- | --- | --- | --- | --- | --- | --- | --- | --- | --- | --- | --- |
| Favilli , 1998(46) | 1 | 1 | 1 | 0 | 1 | 0 | 1 | 1 | 0 | 0 | 0 | 6 |
| Ortiz ,2010（38） | 1 | 0 | 1 | 0 | 1 | 0 | 0 | 0 | 0 | 0 | 0 | 3 |
| Roofthooft ,2011（51） | 1 | 1 | 1 | 1 | 1 | 1 | 0 | 1 | 0 | 1 | 0 | 8 |
| Mat Bah ,2021(24) | 1 | 1 | 1 | 0 | 1 | 1 | 1 | 1 | 0 | 1 | 0 | 8 |
| Jastania ,2022(26) | 1 | 1 | 1 | 0 | 1 | 0 | 1 | 1 | 0 | 0 | 0 | 6 |
| Qian ,2022(27) | 1 | 1 | 1 | 1 | 1 | 0 | 1 | 1 | 0 | 1 | 1 | 9 |

# Supplementary File S6. The Jadad to rate the risk of bias in RCT studies

| **References** | **Randomization** | | | **Concealment of allocation** | | | **Blinding** | | | **Withdrawals and dropouts** | | **Total (max score: 7)** |
| --- | --- | --- | --- | --- | --- | --- | --- | --- | --- | --- | --- | --- |
|  | **Not randomized or inappropriate method of randomization.** | **The study was described as randomized** | **The method of randomization was described and it was appropriate.** | **Not describe the method of allocation concealment.** | **The study was described as using allocation concealment method** | **The method of allocation concealment was described appr 이)riately.** | **No blind or inappropriate method of blinding** | **The study was described as double blind** | **The method of double blinding was described and it was appropriate** | **Not describe the follow-up** | **A description of withdrawals and dropouts** |  |
| Wessel, 1997(31) |  |  | 2 |  |  |  | 0 |  |  |  | 1 | 3 |
| Kinsella, 1997(72) |  |  | 2 | 0 |  |  | 0 |  |  |  | 0 | 3 |
| Davidson, 1998(7) |  |  | 2 |  |  | 2 |  |  | 2 |  | 1 | 7 |
| Wood ,1999(32) |  |  | 2 | 0 |  |  | 0 |  |  |  | 1 | 3 |
| Cornfield, 1999(33) |  |  | 2 | 0 |  |  |  | 1 |  |  | 1 | 4 |
| Clark,2000（8） |  |  | 2 |  |  | 2 |  |  | 2 |  | 1 | 7 |
| Ellington, 2001(10) |  |  | 2 |  |  |  | 0 |  |  |  | 1 | 3 |
| Lipkin,2002(11) |  |  | 2 |  | 1 |  | 0 |  |  | 0 |  | 3 |
| Vargas-Origel,2010(76) |  | 1 |  | 0 |  |  | 0 |  |  |  | 1 | 2 |
| Rohana, 2011(15) |  |  | 2 | 0 |  |  | 0 |  |  |  | 1 | 3 |
| Uslu, 2011(50) |  |  | 2 |  |  |  | 0 |  |  |  | 1 | 3 |
| Mohamed,2012(84) |  |  | 2 | 0 |  |  |  |  | 2 |  | 1 | 5 |
| El-Ghandour,2020 |  |  | 2 |  |  | 2 |  |  | 2 |  | 1 | 7 |
| Abdelkreem,2021 |  |  | 2 |  |  | 2 |  |  | 2 |  | 1 | 7 |
| Imam,2022 |  |  | 2 |  | 1 |  | 0 |  |  |  | 1 | 4 |
